# Association syndrome de Gougerot Sjogren et maladie cœliaque

**DOI:** 10.11604/pamj.2015.22.33.7608

**Published:** 2015-09-15

**Authors:** Naziha Khammassi, Dorsaf Mohsen, Youssef Kort, Haykel Abdelhedi, Ouahida Cherif

**Affiliations:** 1 Service de Médecine Interne, Hôpital Razi, 2010 Manouba, Tunisie

**Keywords:** Syndrome de Gougerot Sjogren, maladie cœliaque, maladies autoimmunes, Gougerot Sjogren Syndrome, celiac disease, autoimmune diseases

## Abstract

De nombreuses pathologies ont été associées à la maladie cœliaque (MC). L'association avec un syndrome de Gougerot Sjogren (SGS) a rarement été rapportée, mais une association due au simple hasard ne peut être exclue. Dans ce cas, le risque d'oncogenèse est double et une surveillance régulière s'impose.

## Introduction

Le SGS est une affection inflammatoire chronique pouvant être primitive (isolée) ou secondaire c'est-à-dire associée à une affection systémique telle que la polyarthrite rhumatoïde, la sclérodermie, la polymyosite, les vascularites, la thyroïdite auto-immune … Le SGJ secondaire à une maladie cœliaque a rarement été rapportée. Nous en rapportons une observation.

## Patient et observation

Il s'agissait d'une patiente de 41 ans est suivie pour une maladie cœliaque depuis l’âge de 8 ans. En Mai 2011 elle a été hospitalisée pour exploration d'une fièvre au long cours associée à une polyarthrite touchant les grosses articulations. Par ailleurs elle rapportait la notion de sécheresse buccale et oculaire évoluant depuis un an. L'examen somatique notait une adénopathie jugulocarotidienne droite de 0,5 cm de diamètre indolore, des adénopathies sous mandibulaires infra centimétriques bilatérales et une polyarthrite touchant les poignets et les chevilles. Le reste de l'examen était sans particularité. A la biologie on notait un syndrome inflammatoire biologique (vitesse de sédimentation à 115 mm la première heure, une CRP à 40 mg/l). Le bilan immunologique avait montré des anticorps antinucléaires positifs avec des anticorps anti SSA, anti SSB et un facteur rhumatoïde positifs. Le «Break up time» (BUT) était altéré à 3’‘ en faveur d'une xérophtalmie. La biopsie des glandes salivaires accessoires était en faveur d'une sialadénite stade IV de Chisholm. La tomodensitométrie thoracoabdominale objectivait des adénopathies interaortico-caves et mésentériques infra centimétriques. Une biopsie d'une adénopathie cervicale a été réalisée et l'examen histologique a conclu à l'absence de signes histologiques de spécificité ou de malignité. La biopsie duodénale montrait une atrophie villositaire totale sans signes histologiques de malignité. La survenue d'un SGS plusieurs années après le diagnostic de la maladie cœliaque et l'absence de toute autre maladie auto-immune nous ont permis de retenir le diagnostic de SGS secondaire à la MC.

## Discussion

La MC, d’étiopathogénie complexe est définie comme une entéropathie sensible au gluten, constituant protéique majeur des céréales, survenant chez des sujets génétiquement prédisposés [1]. Le mode de présentation et les manifestations cliniques de la MC chez l'adulte ont changé de profil. Les manifestations extradigestives peuvent être au premier plan avec des symptômes digestifs discrets voire absents. Le SGS est une maladie auto-immune systémique caractérisée par une infiltration lymphoïde des glandes exocrines, principalement salivaires et lacrymales, responsable d'un syndrome sec. Le SGS peut être primitif ou secondaire à une autre maladie auto-immune dont la MC. Chez notre malade le diagnostic de SGS a été retenu sur des arguments cliniques, immunologiques et histologiques. La MC est associée à un excès d'autres maladies auto-immunes, parfois multiples dans 5 à 30% des cas [2]. Il peut s'agir de maladies auto-immunes spécifiques d'organe (diabète insulinodépendant, thyroïdite auto-immune, cirrhose biliaire primitive, etc …) ou maladies auto-immunes systémiques (lupus systémique, maladie de Gougerot-Sjögren, etc …) Le SGS et la MC présentent plusieurs similitudes cliniques, biologiques et histologiques. Parmi les manifestations extradigestives de la MC, les manifestations articulaires à type de mono- ou polyarthrite séronégative, non érosive et non déformante ont été rapportées chez 7 à 40% des patients avec MC [1, 3]. Sur un plan diagnostique voire pathogénique un lien entre SGS et colites microscopiques.

(colite lymphocytaire) et MC peut être discuté, la MC étant souvent associée au SGS [4]. Le diagnostic formel de MC est établi avec la mise en évidence sur les biopsies duodénales d'une atrophie villositaire totale ou subtotale accompagnée d'une hypertrophie cryptique, et d'une infiltration lymphocytaire de l’épithélium de surface. Un syndrome de malabsorption du grêle reste un tableau classique évocateur de la MC. Cependant il convient d'insister sur l'existence potentielle, au cours du SGS, d'une malabsorption, avec amaigrissement qui s'accompagne fréquemment d'un déficit en oligo-éléments et en vitamines [5]. Celle- ci trouve son origine dans: l'atrophie de l’épithélium intestinal, la présence d'une hypochlorhydrie, d'une atteinte des secrétions pancréatiques et biliaires et d'une pullulation microbienne, comme cela a été démontré dans certaines autres pathologies [6, 7]. En effet, des études récentes ont montré que l'incidence de la MC était dix fois plus fréquente dans le SGS que dans la population générale (4,5/100 versus 4,5 à 5,5/1000). Il est donc possible que le syndrome de malabsorption ne soit pas la conséquence d'un SGS mais celle d'une maladie cœliaque méconnue qui devrait être recherchée par le dosage des anticorps anti-transglutaminases tissulaire dont le rendement diagnostic est bon [1]. Au cours du SGS, il convient de garder en mémoire la possibilité de cancer de l'estomac ou de lymphome malin non hodgkinien. La MC peut aussi se compliquer de lymphome malin non hodgkinien, de localisation le plus souvent digestive et de cancers épithéliaux [8]. Le risque d'oncogenèse est donc double chez notre patiente. Par conséquent une surveillance régulière est ainsi proposée pour dépister cette complication.

## Conclusion

A notre connaissance l'association maladie cœliaque et syndrome de SGS a rarement été rapportée. La survenue d'un cancer au cours de l’évolution de ces deux pathologies impose un suivi régulier.
